# Dynamic interaction of injected liquid jet with skin layer interfaces revealed by microsecond imaging of optically cleared ex vivo skin tissue model

**DOI:** 10.1186/s13036-023-00335-x

**Published:** 2023-02-27

**Authors:** Abdul Mohizin, Jakir Hossain Imran, Kee Sung Lee, Jung Kyung Kim

**Affiliations:** 1 School of Mechanical Engineering, Kookmin University, 77 Jeongneung-Ro, Seongbuk-Gu, Seoul, 02707 Republic of Korea; 2 Department of Mechanical Engineering, Graduate School, Kookmin University, Seoul, 02707 Republic of Korea

**Keywords:** Needle-free jet injection, Transdermal drug delivery, Interfacial interaction, Deep tissue imaging, Near-infrared imaging, Tissue clearing, Skin tissue model

## Abstract

**Background:**

Needle-free jet injection (NFJI) systems enable a controlled and targeted delivery of drugs into skin tissue. However, a scarce understanding of their underlying mechanisms has been a major deterrent to the development of an efficient system. Primarily, the lack of a suitable visualization technique that could capture the dynamics of the injected fluid–tissue interaction with a microsecond range temporal resolution has emerged as a main limitation. A conventional needle-free injection system may inject the fluids within a few milliseconds and may need a temporal resolution in the microsecond range for obtaining the required images. However, the presently available imaging techniques for skin tissue visualization fail to achieve these required spatial and temporal resolutions. Previous studies on injected fluid–tissue interaction dynamics were conducted using in vitro media with a stiffness similar to that of skin tissue. However, these media are poor substitutes for real skin tissue, and the need for an imaging technique having ex vivo or in vivo imaging capability has been echoed in the previous reports.

**Methods:**

A near-infrared imaging technique that utilizes the optical absorption and fluorescence emission of indocyanine green dye, coupled with a tissue clearing technique, was developed for visualizing a NFJI in an ex vivo porcine skin tissue.

**Results:**

The optimal imaging conditions obtained by considering the optical properties of the developed system and mechanical properties of the cleared ex vivo samples are presented. Crucial information on the dynamic interaction of the injected liquid jet with the ex vivo skin tissue layers and their interfaces could be obtained.

**Conclusions:**

The reported technique can be instrumental for understanding the injection mechanism and for the development of an efficient transdermal NFJI system as well.

**Supplementary Information:**

The online version contains supplementary material available at 10.1186/s13036-023-00335-x.

## Introduction

Since the invention of hypodermic needle syringes, injection into dermal compartments has been widely employed for administering liquid-based vaccines, drugs, or other therapeutic agents into the human body. They played a crucial role in the vaccination procedure for COVID-19 and stressed the dependency of humans on a targeted drug delivery system for their survival. As the world became heavily dependent on hypodermic needle-based injection systems that were used in mass vaccination campaigns to eradicate the pandemic, crucial limitations of these systems like needle phobia and biowaste became apparent. To date, needle phobia has remained one of the most important challenges for the mass immunization campaigns organized by the governments of different countries for their citizens. Approximately 11.5 to 66 million adults in the United States suffer from needle phobia [1], and nearly 10% of the population of the United Kingdom resisted the COVID-19 vaccine because of this phobia [2]. This underlines the necessity of an alternative efficient drug delivery system that can overcome these critical limitations.

Needle-free jet injection systems (NFJISs) have been proposed as alternatives for the conventional hypodermic injection systems. At present, the commercially available NFJISs fall into the category of impulse driven systems, in which a moving piston is used to pressurize the fluid in an injection chamber, and as a result, the pressurized fluid is converted into a high-velocity microjet while passing through a micronozzle (nozzle diameter: ~ 50–300 µm [3]). Based upon the actuation type used for the displacement of the piston, they are usually classified as mechanically driven or electrically driven injectors [4]. A compressed spring [5–13] or an expanding gas or air [14–21] is used as the energy sources for mechanical actuators, whereas electrical actuators use the Lorentz force [22–29] or piezoelectric energy [30, 31] to pressurize the injection fluid to obtain high-velocity microjets. These propelled microjets have velocities of approximately 100 m/s, which may be sufficient to penetrate the skin surface (reported to be around 15 MPa [32]) and deliver the drug to a particular depth in the skin tissue. The majority of the current commercially available injectors use mechanically actuated systems like compressed air or pressurized springs to energize the piston, and thus, lack dynamic control, which is a major limitation of these systems. In such cases, once the injection parameters are set, the penetration and dispersion characteristics of the propelled microjet cannot be changed or controlled. This limits the applicable jet velocity and volume of the system [33], and thus, such systems cannot adapt to variations in the mechanical properties of skin [30]. If the initial injection setting results in a low propelled velocity, then the fluid may not penetrate the dense superficial layers, and if the propelled velocity is too high, then the penetration depth may be too large and also significant splash-back of the injected fluid could occur resulting in a poor delivery [30]. Pain and bruising associated with the injections are mainly because of this uncontrollability of the injection parameters, which is the major limiting factor of the current version of the NFJISs.

However, NFJIS has the potential to be a better targeted and controlled drug delivery technique as it could achieve a wide range of penetration depth in the skin matrix, and as a result, can target various dermal compartments for an effective drug delivery. Recent advancements in small volume needle-free jet injections and dynamic control mechanisms could unlock the superficial layers of the skin like dermis and epidermis, and are anticipated to have a tremendous impact on the current drug delivery technology [33]. Nevertheless, the ideal penetration profile or the required microjet velocity profile for achieving it remains hitherto unknown. The mechanism in an NFJIS poses a unique problem, as the injected drug as microjet has to penetrate through various layers of the skin tissue and has to interact with these layers and their interfaces during the penetration and dispersion phases. Further, the typical injection pressure encountered in NFJISs is usually in the MPa range, whereas that in the conventional hypodermic injection systems is typically in the kPa range [34]. Thus, understanding the injected fluid–tissue interactions is critical for the development of an efficient NFJIS.

Many researchers have reported the engineering and clinical aspects of NFJISs [3, 6, 7, 11–13, 17–19, 21, 24, 31, 35–46]. However, the exact mechanism or the influence of various skin layers and their interfaces on the injection characteristics is still unknown. A major factor that hinders the investigations of the underlying mechanism is the lack of visualization techniques that can capture the entire injection process inside the skin tissue with required spatial and temporal resolutions. A spatial range of several millimeters is required to visualize the entire injection profile, and the required temporal resolution is ~ 50 μs for visualizing the microjet penetration into the skin tissue and about 1–2 μs for observing the penetration through the epidermis layer. However, the maximum attainable temporal resolution in the present imaging techniques for skin tissue visualization is about 1.5–2 ms [47], which is not adequate for the real-time visualization of a fluid injected by an NFJIS into the skin tissue matrix. Hence, in majority of the studies, in vitro media like agarose, gelatin, or polyacrylamide gels [6, 7, 13, 21, 36, 38, 41, 43, 45, 46, 48–55] were used for investigating the dynamic injection characteristics of an NFJIS, while the ex vivo studies were performed via dissection after the injection [10, 11, 15, 37, 38, 56–59] or by micro-computed tomography [9] or X-ray [38, 60] imaging techniques. In vitro models are indispensable tools in drug delivery applications for investigating the effect of individual parameters and to tune the independent and alternative parameters. Additionally, they are popular in studying NFJISs owing to their transparency, which facilitates the investigation of the interaction of the injected high-velocity microjet with the surrounding media. Based on these studies, it is hypothesized that for a large volume microjet injections, the fluid penetrates up to a particular depth without much diffusion, after which it may remain stagnant for some microseconds. This depth is termed as the initial penetration depth, and a subsequent influx of the fluid causes the fluid to be dispersed radially outward in a near spherical manner [37, 40, 42, 43, 57, 61]. The penetration depth achieved at the end of the injection is termed as the final penetration depth. However, this hypothesis is based on homogeneous in vitro media, and does not incorporate the anisotropic nature of the skin tissue matrix, which comprises multilayers. The varying property of the tissue layers and their interfaces play a crucial role in the injection characteristics of an NFJIS. The reported penetration depth in the in vitro media may be significantly higher than that of the actual skin, because these models are based on the Young’s modulus parameter alone and have limited complexity compared to a skin tissue matrix. The limitations of considering the Young’s modulus as the sole mechanical parameter of a medium for an NFJIS is discussed in detail in our previous report [46]. These in vitro media need to be modified to incorporate additional parameters like pore size and fracture toughness to replicate a layer of skin tissue in an engineering perspective. Thus, an advanced imaging technique for both ex vivo and in vivo visualization of the injection characteristics is the first and foremost requirement for the development of a targeted controlled delivery system like NFJISs.

Herein, a near-infrared (NIR) imaging technique coupled with a skin tissue clearing technique was developed to achieve high temporal and spatial resolutions in a porcine ex vivo skin tissue for visualizing an NFJI. The NIR-I window (650–900 nm wavelength) imaging technique was used to achieve a light penetration into deeper tissue, made possible due to the low scattering and absorption of light at this wavelength band by blood and hemoglobin [62]. Moreover, the autofluorescence of skin, fat, and blood is significantly lower in the NIR-I range than in the visible light range [63]. Thus, a high-sensitivity and high-resolution imaging can be realized in the NIR-I region because of the high signal-to-background ratio (SBR) in tissue samples. Additionally, the NIR radiation is nonionizing and thus poses no risk of tissue damage or genotoxicity [62, 64]. However, the maximum imaging depth into the skin is limited with the NIR-I imaging mode alone for the required spatial and temporal resolution. A temporal resolution of about 200 ms was reported in literature for achieving a deeper imaging depth in NIR range [65, 66] and thus is not suitable for the present investigation. In the present study, indocyanine green (ICG) dye illuminated with a 785 nm wavelength light was used to obtain a sufficient contrast of injection profile with the surrounding tissue and the imaging was done with fluorescence and absorbance modes. In the fluorescence imaging mode, the fluorescence signal emitted by the ICG dye excited at the NIR-I window was detected, whereas in the absorbance imaging mode, the differences in the light absorption and scattering properties of the dye and tissue medium were exploited for achieving the necessary contrast. From the preliminary study it was evident that a satisfactory contrast and SBR could be achieved up to a imaging depth of ~ 2 mm into the skin tissue; which was not suitable for visualizing the injection profile in the skin tissue matrix. Hence, an immersion-based skin tissue clearing technique was used to improve the imaging depth into the skin matrix. Clearing time by immersion into a 60% 2,2’-thiodiethanol (TDE) solution was optimized by considering the optical and mechanical properties of the cleared tissue. Clearing by TDE is a simple protocol and is considered harmless, non-hazardous, and easy to handle [67]. TDE is commercially available and inexpensive, and the protocol can be readily performed in laboratories without any specialized training, making it ideal for the engineering and clinical research groups investigating the characteristics of NFJISs.

An NFJI in an ex vivo porcine skin tissue was visualized with the optimized imaging conditions obtained from the preliminary studies, and it was observed that the applied techniques could clearly identify the various phases in the needle-free injection, such as the penetration, dispersion, and post-injection dispersion phases distinctively. To the best of our knowledge, this is the first time an NFJI has been visualized in an ex vivo tissue sample at a frame rate > 10,000 fps. The analysis of the acquired images provided crucial insights into the dynamic interaction of the high-velocity microjet with the skin tissue and could be a key tool in understanding the underlying mechanism of an NFJIS. Knowing the dynamic interactions of the injected fluid with the skin tissue layers and their interfaces are crucial for optimizing the delivery characteristics to achieve the required therapeutic effect. Without this knowledge, the injected drug may not reach the targeted region nor can penetrate further into the subsequent layer and thereby restrict the availability of the drug for achieving the required therapeutic effect.

## Results and discussion

### Optimal imaging considerations

#### Optimization of exposure time and laser power

**Fig. 1:**
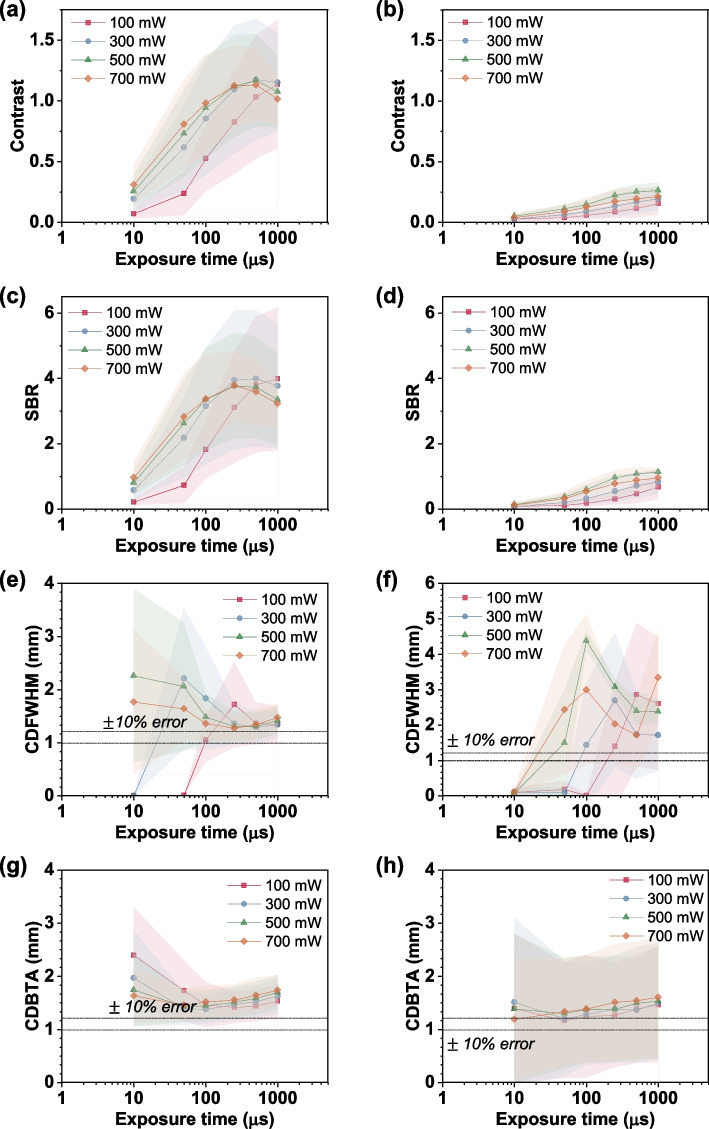
Fluorescence imaging data of the non-cleared ex vivo tissue samples. (**a**) Contrast (**c**) signal-to-background ratio (SBR) (**e**) capillary diameter measured by full width at half maximum (CDFWHM), and (**g**) capillary diameter measured by binarization and thresholding algorithm (CDBTA) measured for P1. (**b**) Contrast (**d**) SBR (**f**) CDFWHM, and (**h**) CDBTA measured for P2

First, we identified the least possible exposure time and other imaging conditions for visualizing deep-tissue injection with minimum errors by inserting capillary tubes filled with the ICG dye at positions P1 (~ 2 mm depth) and P2 (~ 4 mm depth) and imaging them at the fluorescence and absorbance modes, respectively. In the fluorescence imaging mode, the ICG dye was excited using a 785-nm light, and the resulting light signals emitted by the dye were collected. To detect only the fluorescence signals, a bandpass filter was used. The incident laser power was increased to realize high-speed imaging of an ex vivo sample. Increasing the incident laser power can reduce the scattering, which can further increase the light penetration depth [68]. Figure 1 shows the obtained quantifiers for the non-cleared tissue under fluorescence imaging conditions. In P1 case, the SBR and contrast increased with the increasing laser power for 10, 50, 100, and 250 µs of exposure. However, at 300, 500, and 700 mW laser power with 500 and 1000 µs of exposure time, the SBR and contrast decreased because of emission signal saturation.

Compared with the P1 case, the P2 case involved a larger scattering effect. The scattering conducts light dispersion in the tissue, ultimately resulting in decreasing energy density with depth [69, 70]. Due to increased imaging depth, and consequently, the SBR and contrast reduced significantly. As a result, the capillary diameter measurements by CDFWHM and CDBTA had higher deviation for P2 compared with the P1 case. This may suggest that the current imaging conditions are not suitable for imaging at an imaging depth > 2 mm.

**Fig. 2:**
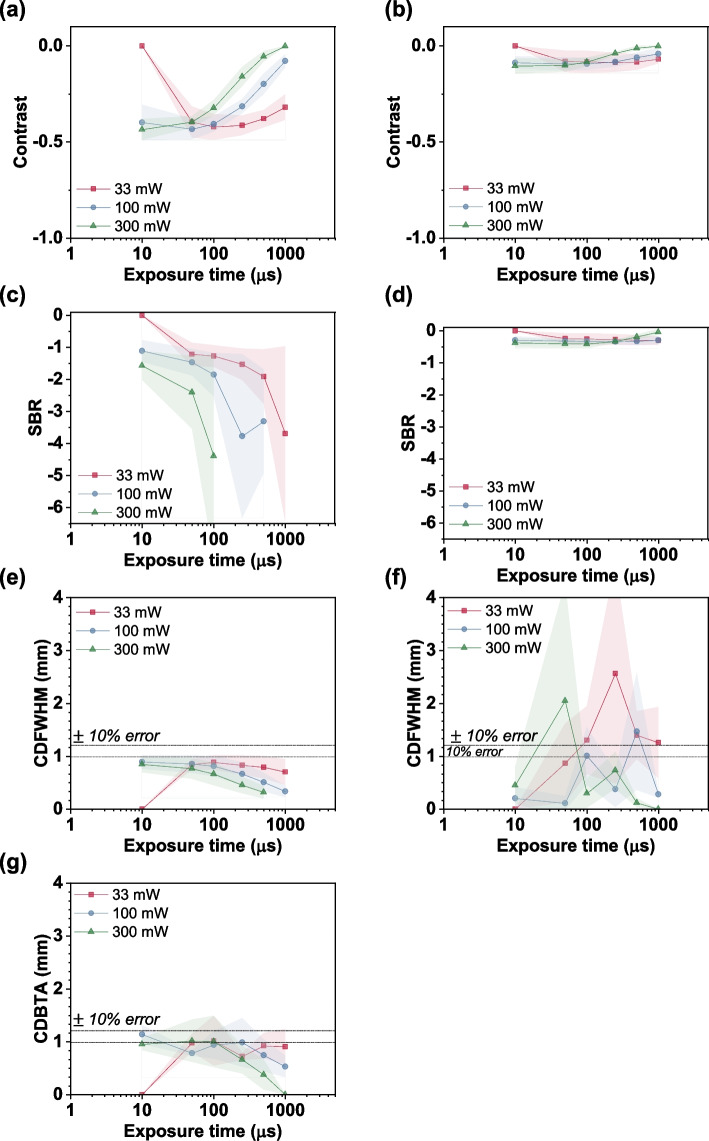
Absorbance imaging data of the non-cleared ex vivo tissue samples. (**a**) Contrast, (**c**) SBR, (**e**) CDFWHM, and (**g**) CDBTA measured for P1. (**b**) Contrast, (**d**) SBR, and (**f**) CDFWHM, obtained for P2. No meaningful CDBTA data could be extracted for P2

In the absorbance imaging mode, the higher absorption of light by the ICG dye compared with that by the skin tissue was utilized. When light passes through a skin tissue tainted with the ICG dye, the ICG dye absorbs the 785-nm light and appears darker than the surrounding medium. Contrast and SBR have negative values for absorbance imaging as the surrounding tissue appears brighter compared to the required signal, and thus could be utilized for identifying and tracing the boundary of the required profile inside the skin tissue. Here the contrast and SBR show higher negative values at P1 than P2. As compared with the fluorescence imaging condition, low laser power was required for achieving the necessary contrast in the region of interest and at high laser power and high exposure time, the images got saturated resulting in a low contrast and SBR quantifiers. Thus, this imaging mode was suitable for low exposure conditions and increasing the laser power improved the image quality at these low exposure conditions. However, the incident laser power was limited to 300 mW in the present imaging conditions to prevent any damage to the camera sensors as no filters were used. Capillary diameter measurements were made with reasonable accuracy for P1 case in non-cleared sample. However, no significant contrast could be achieved between the capillary tube and the surrounding tissue for the P2 case, and thus, no measurable signal could be obtained in this case. This may be due to the increased scattering of light with the increase in the imaging depth. Hence NIR imaging alone could not be applied effectively for deeper imaging depth than 2 mm. Figure 2 depicts these imaging quantifiers for absorbance imaging mode.

**Fig. 3:**
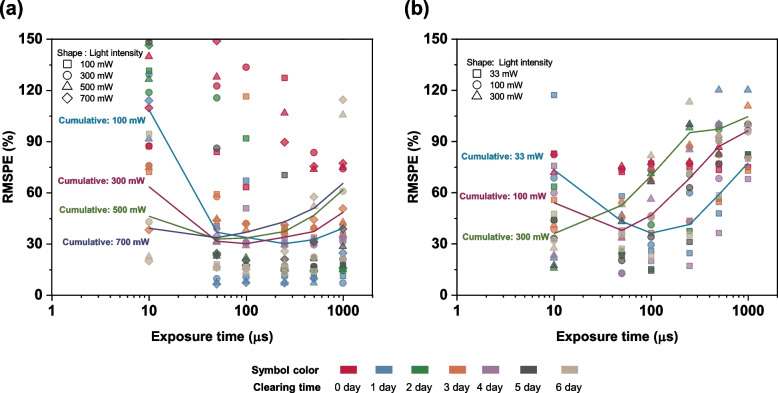
Root mean square percentage error (RMSPE) measured for P1 and P2 under (**a**) fluorescence and (**b**) absorbance imaging modes. Cumulative error for each laser power under different clearing times is shown using the thick line

To improve the visualization capability for depths deeper than 2 mm, tissue clearing was performed with 60% TDE for up to six days. Significant improvement in the imaging quantifiers was observed during the tissue clearing. The exposure time, laser power, and clearing time were measured for identifying the optimal imaging condition. Exposure time and laser power are purely optical imaging conditions, but the clearing time is obtained by considering the changes in the mechanical and optical characteristics of the tissue during the process. Hence, the exposure time and laser power were deduced by considering the cumulative error in the measured capillary diameter during all the imaging cases. The root mean square percentage error (RMSPE), which yields the cumulative error in the capillary measurement for a certain exposure time and a laser power for all the clearing times, was used to obtain the optimum parameters. Figure 3 shows the variation in RMSPE with exposure time and laser power. The optimum parameters for fluorescence imaging were determined as 50 μs (exposure time) and 500 mW (laser power), while those for the absorbance imaging mode were found to be 10 μs and 300 mW.

#### Optical imaging considerations with clearing time

**Fig. 4:**
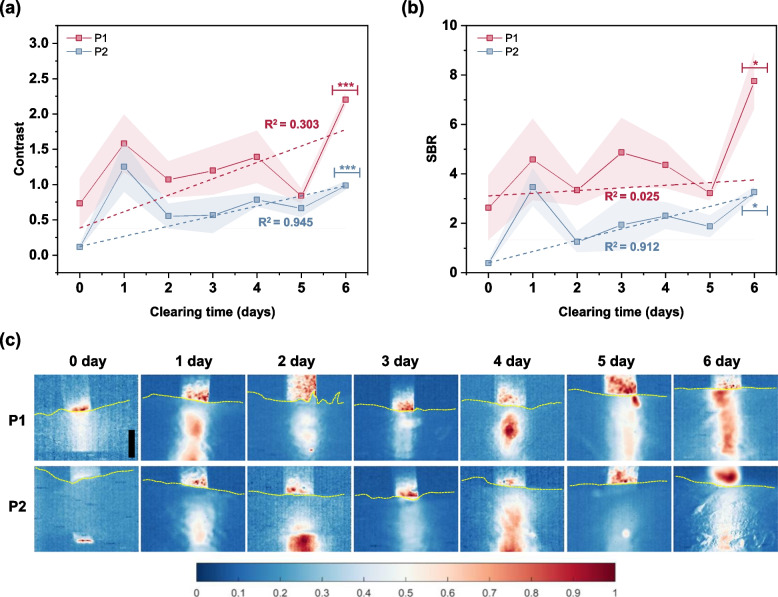
Variation of (**a**) contrast and (**b**) SBR with clearing time in the fluorescence imaging mode. Data series marked by (***) and (*) have *p*-value $\le$ 0.001 and *p*-value $\le$ 0.05, respectively. **c** Qualitative visualization of the capillary tubes under fluorescence imaging mode on a normalized scale. Scale bar = 1 mm. Skin tissue surface is marked by the yellow dashed lines

**Fig. 5:**
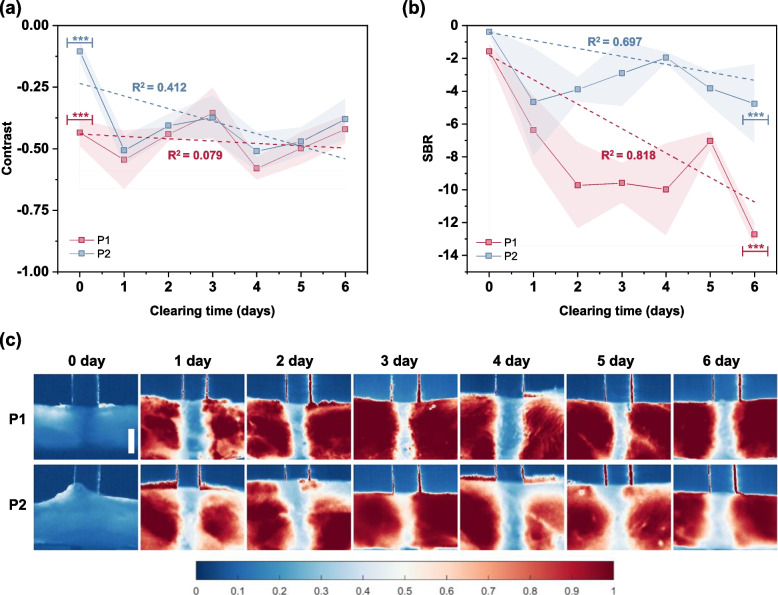
Variation of (**a**) contrast and (**b**) SBR with clearing time in the absorbance imaging mode. Data series having a *p*-value $\le$ 0.001 are marked by (***). **c** Qualitative visualization of the capillary tubes under absorbance imaging mode on a normalized scale. Scale bar = 1 mm

The variations in the optical quantifiers with the clearing time at 50 μs of exposure time and 500 mW laser power (for fluorescence imaging) and at 10 μs of exposure time and 300 mW laser power (for absorbance imaging) are plotted in Figs. 4 and 5, respectively.

The contrast and SBR in both fluorescence and absorbance imaging modes had a zigzag profile and were higher in 2 mm depth (P1) compared with the 4 mm depth (P2) at different tissue clearing times except for the contrast at 3 day clearing time in absorbance imaging mode. This deviation could be considered as an outlier and may have been due to the dynamic interaction of optical clearing agent (OCA) with the skin tissue causing variations in its optical properties.

Evidently, the contrast and SBR increased with the increasing clearing time for the fluorescence imaging, while they decreased for the absorbance imaging. Theoretically, by applying tissue clearing technique, the tissue transparency is enhanced by the reduction of light scattering [71]. However, in the present study, the samples are rich with collagen fibers interacting with the OCA that can alter the optical and mechanical property of the tissue, which was varying in a nonlinear manner with the clearing time. The mechanism of interaction between the OCA and the skin tissue sample is beyond the scope of this work and detailed study may be needed to investigate the interaction. In general, it could be summarized that the transparency of the sample improves with the increase in clearing time, resulting in better imaging conditions for both the fluorescence and absorbance imaging.

**Fig. 6:**
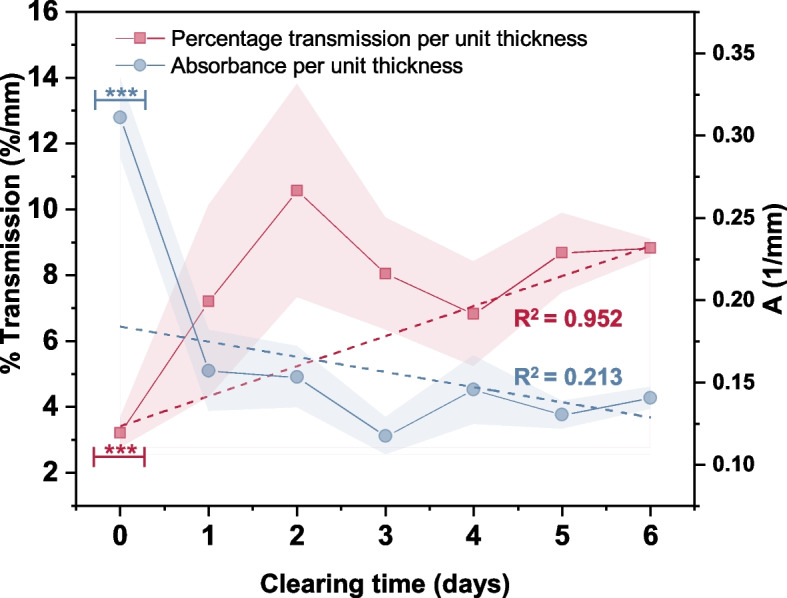
Impact of clearing time on light transmittance and absorbance. Symbol (***) denotes a *p*-value $\le$ 0.001

To validate the observation, the optical characteristics of the skin tissue samples were measured for various degrees of optical tissue clearing. Figure 6 shows the normalized absorbance and transmission of a 785-nm light incident on the skin tissue samples, indicating that the transmission coefficient increased with the increasing clearing time, whereas the corresponding absorbance coefficient decreased with the increasing clearing time. Thus, it can be inferred that the light transmission through a cleared tissue may increase with the clearing time in 60% TDE, which is favorable for obtaining high-quality images for the present application. However, the mechanical parametric effects during tissue clearing should also be considered while obtaining the optimum clearing time for the present application.

### Mechanical property considerations

#### Shrinkage

**Fig. 7:**
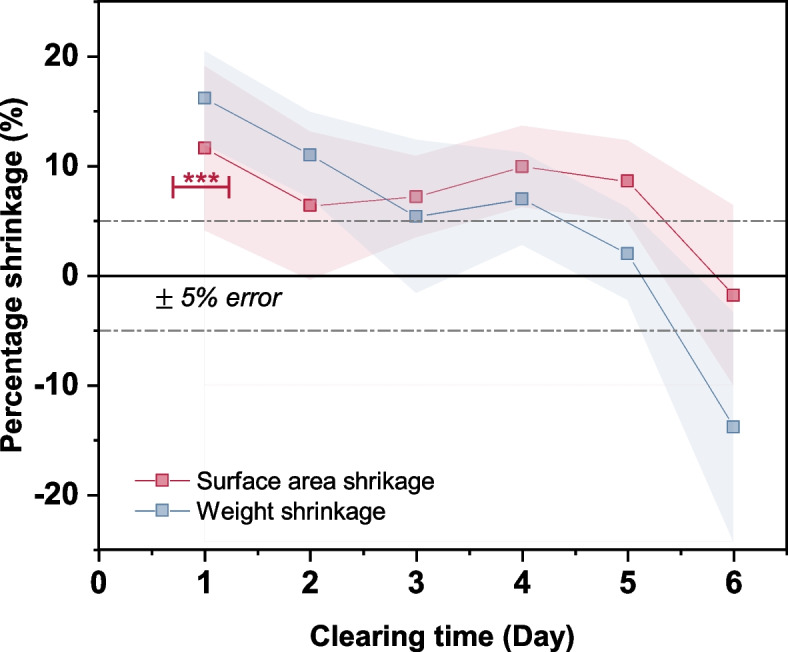
Percentage shrinkage of surface area and weight measured over various clearing times. Error band shows the standard deviation of 10 trials. (***) signifies a *p*-value $\le$ 0.001

Shrinkage of tissue samples occurs in majority of the optical tissue clearing techniques and is an undesirable phenomenon [72]. We quantified the dimensional and weight variations of the skin tissue samples during the tissue clearing process, and the corresponding results are presented in Fig. 7. Tissue shrinkage occurred when the sample was immersed in the TDE solution, possibly because optical clearing by TDE includes dehydration.

Typically, TDE is used as a refractive index (RI) matching OCA [73]. It is speculated that the difference between the RI of the lipid bilayer membranes and that of the cytoplasm is reduced by the chemical action of the reagent, resulting in the reduction of light scattering in the sample [67]. However, in the present study, the sample under investigation was a skin tissue with a dermis layer, which is rich in collagen fibers that strongly scatter light in biological tissues [74]. When alcohol-based OCAs are used for tissue clearing, the hydroxyl groups present in the OCAs interact with these collagen fibers, resulting in better optical clearing efficacies with higher relative transmittances [73, 75].

In such a scenario, optical clearing by immersion in a hydrophilic OCA could occur via three basic steps: dehydration, dissociation of collagen, and RI matching [73]. Our observations also suggest that such a process could have occurred. Due to the high concentration gradient between the OCA and the skin tissue, water may have been replaced by the OCA in the skin tissue samples initially, and the highest percentage variation was observed for the sample with one-day tissue clearing. With the passage of time, the concentration difference decreased and the expelled water was reabsorbed by the skin tissue. Thus, the dimensional and weight shrinkage percentages of the sample decreased, and after five days, the sample shrinkage was the lowest, suggesting that the lost moisture content was reabsorbed by the skin tissue samples. Further absorption of water molecules from the solution may have resulted in the observed sample swelling after six days of clearing.

#### Variation of Young’s modulus and fracture toughness

Young’s modulus and fracture toughness of the injection media play crucial roles in the penetration and dispersion characteristics of a needle-free jet injection. However, only limited studies investigated their influence. Studies by Shergold and Fleck [76] and Scramm-Baxter et al. [43] used in vitro media to investigate the impact of Young’s modulus and observed that the penetration depth of the microjet decreased with the increase in Young’s modulus. Shergold and Fleck [76] described the role of fracture mechanism when the liquid jet penetrated the skin and proposed prediction models by considering the penetrating microjet as a flat and sharp tipped punch, which agreed with the experimental result [32]. In our previous report [46], we have emphasized the importance of fracture toughness on the penetration and dispersion characteristics of a needle-free jet injection process and proposed a hypothesis that there exists a critical fracture toughness parameter that influences the penetrating capability of the microjet. It has been reported that the fracture toughness of the in vitro hydrogel [77] media is on the order of 10 J/${\mathrm{m}}^{2}$ whereas that of the skin tissue is on the order of ${10}^{3}$ J/${\mathrm{m}}^{2}$ (2500–4100 J/${\mathrm{m}}^{2}$) [32, 78, 79].

**Fig. 8:**
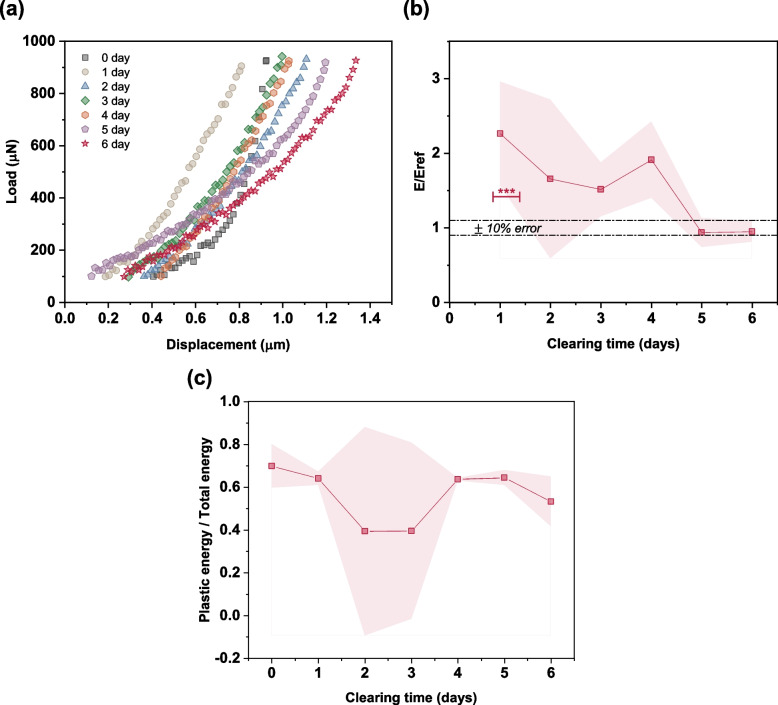
**a** Load profile of the ex vivo porcine skin tissue sample under different clearing times. **b** Relative variation of Young’s modulus with clearing time in TDE. E_ref_ is the Young’s modulus of non-cleared tissue sample. Standard deviation of three trials is shown as error band. **c** Ratio of plastic energy to total energy during indentation study

Effect of tissue clearing time with 60% TDE on the mechanical property of the sample is plotted in the Fig. 8a and b. The loading profile of the non-cleared tissue sample was non-linear. However, because of the OCA action, a linear profile was obtained for up to five days of clearing time, after which the profile became non-linear again in the loading range. Further, the relative Young’s modulus variation was within 10% error limit after the fifth day of clearing. This observation was consistent with the shrinkage observation, indicating that because of the loss of moisture, the skin tissue samples became stiffer. However, the reabsorption of water molecules enabled the stiff tissue sample to regain its elasticity property.

Fracture toughness can be defined as the energy that a sample can absorb until it deforms or fractures and requires specific experimental techniques to obtain it. However, it has been proposed that the indentation energy per unit contact area until reaching a critical indentation depth can be related to the fracture energy of ductile materials [80]. Thus, the energy terms associated with the indentation test could be used as quantifiers to compare the fracture toughness parameter [80, 81]. Herein, we compared the ratio of the plastic energy to the total energy obtained for various degrees of tissue clearing and found a negligible variation (Fig. 8c). This suggests that there may not be a significant deviation in the fracture toughness parameter of the specimens.

### Ex vivo visualization of NFJI

**Fig. 9:**
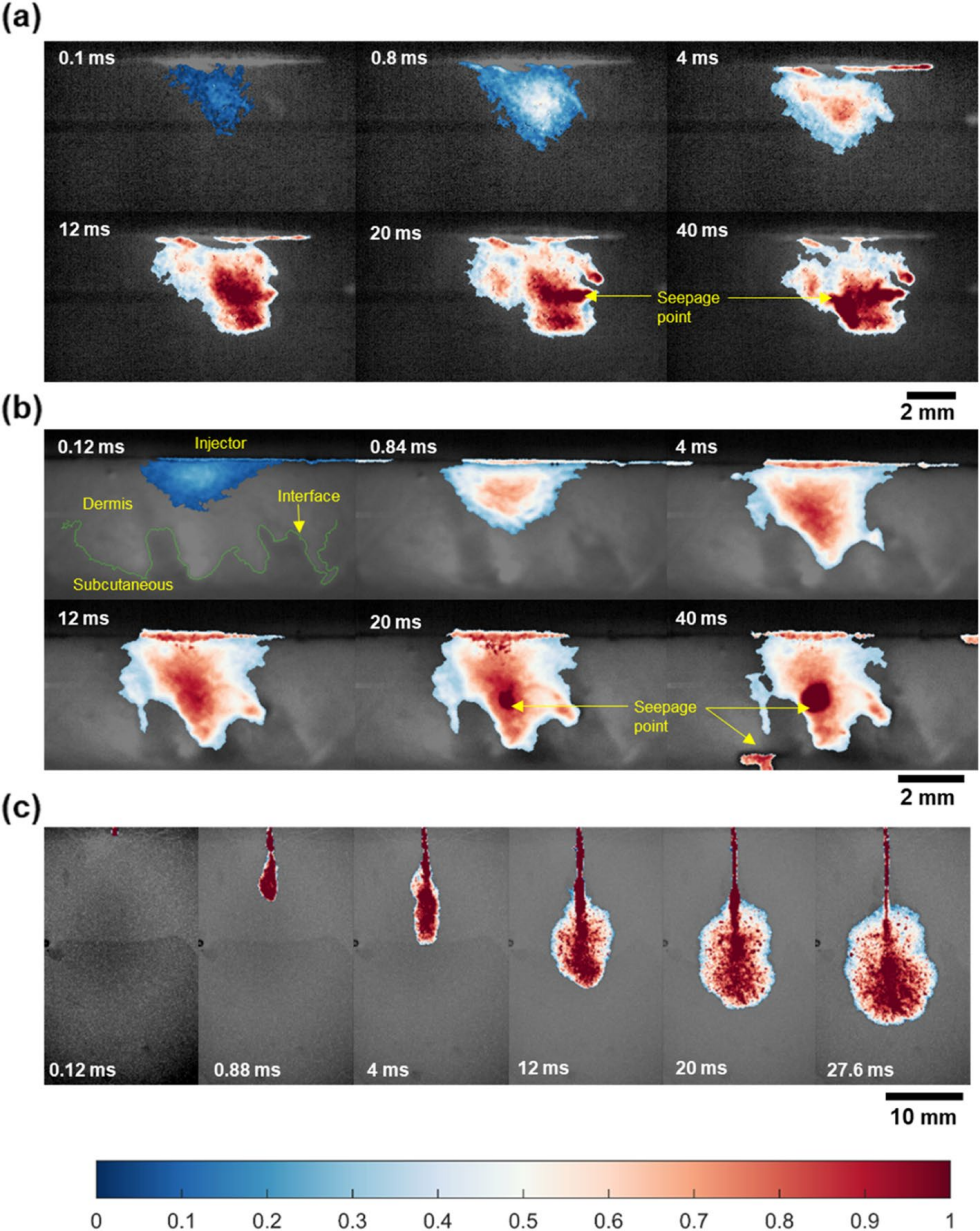
Needle-free jet injection visualization in ex vivo porcine skin tissue under optimum (**a**) fluorescence imaging and (**b**) absorbance imaging conditions. Color bar signifies the relative presence of fluid based on pixel intensity. For fluorescence imaging mode, pixel intensity in the injection front was higher than surrounding and it was lower for absorbance imaging mode. The variation was normalized into a uniform scale to show the relative presence of injected fluid inside the tissue. **c** The penetration profile of the injected fluid in 15% polyacrylamide hydrogel

**Fig. 10:**
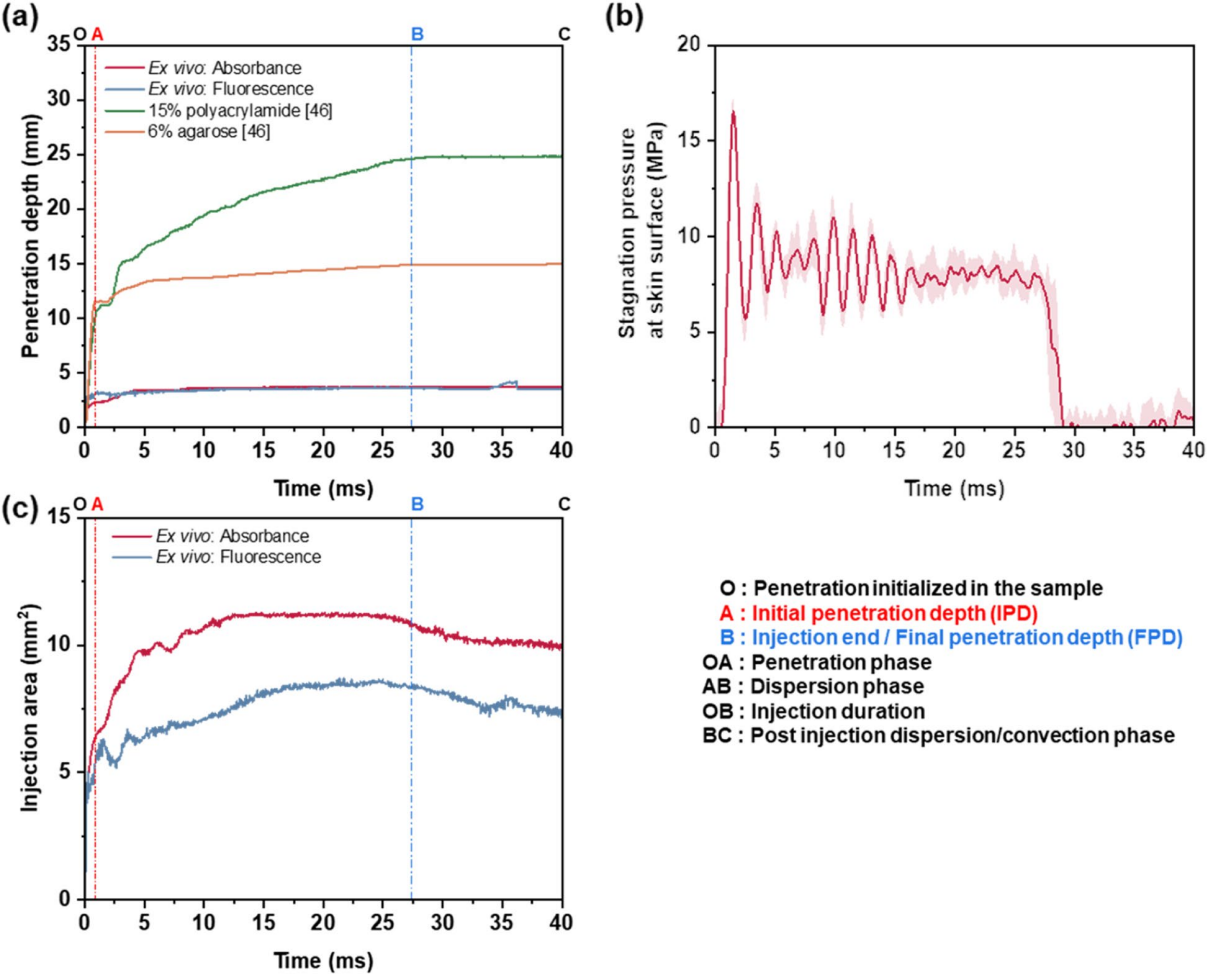
**a** Initial penetration depth data obtained from the ex vivo visualization compared with those reported previously in polyacrylamide and agarose hydrogels [46]. **b** Microjet stagnation pressure data of the injector obtained at 100 µL injection conditions. **c** Variation of projected area of the injection front visualized in the fluorescence and absorbance imaging modes. Various phases of the injection are marked as well

Based on the presented data, the optimum imaging conditions were found to be 50 μs (exposure time) and 500 mW (laser power) for fluorescence imaging and 10 μs and 300 mW for absorbance imaging. Further, with mechanical property considerations, the optimum clearing time for the present application, with 60% TDE, was deduced to be five days. The NFJI visualization was performed under these imaging conditions, and the corresponding results are presented in Fig. 9. We injected 100 μL of fluid using a commercially available injector named Comfort-in, to examine the dynamic interaction of the high-velocity microjet with the skin tissue matrix. In the fluorescence imaging mode, the injected fluid was imaged with a black background (Fig. 9a), whereas in absorbance imaging, the skin tissue layers were clearly distinguished with various pixel intensities, and the injected fluid was imaged with a high contrast (Fig. 9b). Moreover, the penetration and dispersion profiles were clearly visible. The penetration profile of the injected fluid was significantly different from that of the homogeneous hydrogel media, qualitatively represented in Fig. 9c and quantitatively in Fig. 10a.

In Fig. 10a, the region OA marks the penetration phase. In this phase, the injected fluid penetrates without much dispersion into the skin tissue and may remain stagnant for a few milliseconds. The displacement at which the velocity of the penetration front first became zero was termed as the initial penetration depth and it occurred around 0.8–0.9 ms in the hydrogels and the ex vivo media. The initial penetration depth was much shallower in the ex vivo media compared to the in vitro hydrogel media and could be mainly due to the difference in the fracture toughness and other property deviations as the skin tissue is made of anisotropic layers with different interfacial properties as compared to the homogeneous in vitro media.

Region AB marks the dispersion phase and was significantly different compared to that in the in vitro media. It has been inferred that the fluid disperses in a near spherical manner after reaching the initial penetration depth (Fig. 9c) [37, 40, 42, 43, 57, 61]. However, the acquired images (Fig. 9a and b, Supplementary video 1 and 2) showed an inverted hemispherical dispersion profile until the injection front reached the dermis–subcutaneous interface. When the injection front first reached the dermis-subcutaneous interface, further penetration into the subcutaneous layer was stopped as if the interface offered resistance. Further influx of the injected fluid caused the injection front to develop along the interface, thereby containing itself in the dermis layer. However, toward the end of the injection, fluid started to seep out of the ex vivo sample through some seepage points. Interestingly, the absorbance images showed that majority of these seepage points were located along the dermis-subcutaneous interface. Although seepage of the injected fluid through the tissue was observed under both imaging modes, in fluorescence imaging, the seepage location could not be identified as the skin tissue being invisible under this imaging modality. The fluid seepage along the tissue possibly occurred because of the influence of the loose interface between the dermis and subcutaneous layers, which offers a low resistance to the fluid flow.

The impact of interfacial properties on the penetration and dispersion was demonstrated in our previous studies using in vitro models [45, 46], which considered the interface to be either a tight (high resistance to flow) or loose interface (low resistance to flow). However, in the present study the interface initially offered higher resistance to flow as if a tight interface, which caused the injection profile to disperse along the interface and subsequently provided lower resistance to flow which caused the formation of seepage points along the interface. Thus, it could be inferred that initially the interface offers a higher resistance to flow, and as the injection progresses the pressure buildup along the interface may increase, causing it to delaminate. This suggests that a critical pressure exists that could change the interfacial resistance of the skin tissue. The seepage points were observed toward the end of the injection, and after the injection process was over, the injection front began to decrease due to the combined effect of backsplash and seepage of the injected fluid as the skin tissue started to relieve the stress built up during the injection. The final penetration depth showed minimal deviation during this post injection dispersion phase (region BC). On the contrary, overall projected area of the injection front (Fig. 10c) showed a decreasing trend towards the injection, suggesting that the fluid was being driven out of the ex vivo tissue sample.

The penetration depth profiles (Fig. 10a) for the absorbance and fluorescence imaging were of similar nature. However, it should be noted that the injection profile was not symmetric in nature, especially when the injected fluid interacted with the skin tissue interfaces (Fig. 9b). The injection bolus takes the path with the least resistance and this could be reflected in the projected area presented in the study. Thus, a deviation was observed for the projected area of the injection front during the dispersion phase AB. A 3-dimensional imaging technique may be needed to visualize these complex interactions and further studies are required to gain a complete understanding of the injected drug–skin tissue interface interaction dynamics.

As shown in Fig. 10b, the average stagnation pressure of the injected fluid was about 8 MPa, which was much higher than that of a normal microneedle injection (order of 100 kPa [34]). Such a high pressure should aid in the convective transfer of the injected drug inside the skin tissue matrix. However, as the timescale of the injection is much smaller than a second, the reactive stress built up inside the skin tissue expels a portion of the injected fluid as backsplash. If the injected drug could delaminate the interface and then disperse along the interface, then the stress built up in the dermis layer can be relieved, which can reduce the amount of backsplash during a needle-free injection and increase the efficiency of the injection process.

### Limitations of the study

The present study focused mainly on achieving the required spatial and temporal resolutions for visualizing an NFJI in an ex vivo skin tissue. Thus, an NIR imaging technique coupled with optical tissue clearing with TDE was employed to achieve the objective. Imaging and clearing parameters were selected by considering the optical and mechanical characteristics of the injected media. Optical tissue clearing involves dynamic interaction of the OCA and the ex vivo medium, which made the property value oscillate depending on the clearing duration. Further studies are required to clearly understand the underlying process and how these dynamic interactions at molecular level impact the mechanical and optical properties. The optical clearing process may change the ex vivo tissue properties to an extent and we tried to limit the deviation in the Young’s modulus and dimensions of the tissue, while improving the optical visibility inside the tissue. Even with considering the deviation, cleared ex vivo tissue can be considered as the best alternative to the in vitro hydrogel media used in the previous NFJI studies.

Absorbance and fluorescence imaging techniques were used in the present study for visualizing the dynamic interaction of the NFJI with the skin tissue media. When a fluorescence substance is activated by a specific wavelength of the light in fluorescence imaging, the substance absorbs the light at that wavelength before emitting light at a longer wavelength. As a result, the energy of the emitted light is lower than the absorbed energy. Thus, to detect the fluorescence signal, we have to use a higher laser power and a longer exposure time compared to the absorbance imaging mode. When using a camera system with poor performance, it can be challenging to be noticed if the emitted signal is too weak.

In absorbance imaging, the pixel intensities of the obtained image depend on the light interaction with the tissue layers and the injected fluid, which may vary from image to image. We tried to improve the data extraction by processing the image by normalization and histogram equalization. However, the accuracy of the reported results can be improved further by considering a more advanced image processing technique, as the segmentation and thresholding were obtained by considering the global parameters. A more sophisticated technique involving deep learning algorithms can extract richer information from the obtained images.

An uncertainty of ± 1 pixel (80 − 90 µm) is to be expected in the dimension measurement from the segmented images. The measured errors for the optimum conditions in the fluorescence imaging at P1and P2 positions in tissue were 6.9% and 16.8%, respectively. In the absorbance imaging, they were 17.1% and 20.7% for P1 and P2 measurements, respectively.

## Conclusions

We addressed one of the most critical challenges encountered in controlled drug delivery via a NFJI, viz. the dynamic visualization of the injection profile in the skin tissue. The development of efficient NFJISs has been hindered by the unavailability of a visualization technique with spatial and temporal resolutions required for studying the interaction of high-velocity microjets with the skin tissues. Thus, we developed an NIR imaging technique, coupled with a tissue clearing technique, to visualize ex vivo skin tissue samples and evaluate the injection characteristics of an NFJIS. The visualization parameters were optimized based on the optical and mechanical quantifiers. The tissue samples were cleared in 60% TDE for five days to acquire the required transparency and to ensure a minimum variation in the mechanical property. The injections performed on the five-day tissue cleared samples resulted in excellent contrast between the injected fluid and the surrounding tissue, and various phases of the NFJI could be clearly identified. Furthermore, the images gave clues on how the injected fluid interacts with an interface and this information can be used for designing an efficient NFJIS. The demonstrated imaging technique could mark a paradigm shift in the high-speed drug delivery applications like NFJIS studies, where a transition from transparent in vitro media to ex vivo media is possible to obtain realistic information to develop a device with targeted and controlled drug delivery capability.

## Materials and methods

### NIR imaging system

We designed experiments with porcine skin tissue to achieve our objectives. The ICG dye (21980-100MG-F, Cardiogreen, Sigma-Aldrich, St. Luis, USA) was used for this experiment because of its popularity in intradermal and subcutaneous administrations for fluorescent imaging studies [82]. ICG in ethanol was reported to have a higher absorption and emission than ICG in water [83, 84]; thus, 1 mg/mL of ICG in ethanol was used (saturated solution).

The imaging system comprised a 785-nm laser diode (MLL-III-785–2.5 W, CNI Laser, Changchun, China) for illuminating the samples. The images were captured using a high-speed video camera (Phantom VEO E-310L, Vision Research, Wayne, NJ, USA). The peak absorption and emission spectra of ICG in ethanol were reported to be 787 and 811 nm, respectively [85]. The camera was placed in-line with the sample. A micro NIKKOR lens (AF-S VR 105 mm f/2.8G, Nikon, Tokyo, Japan) was attached with the camera to obtain a wide field of view imaging. The laser source was placed near the sample at an incident angle of approximately 15°. It would be ideal to locate the laser source normal (incident angle of 0°) to the sample in order to obtain a uniform light illumination. Non-uniform illumination can cause low quality images and unusable images in some cases, especially when the variation of illumination exceeds the sensitivity range of the camera. The smallest incident angle that we could obtain was 15° due to camera position. Therefore, the laser beam was projected on the ICG loaded capillary tube to excite the ICG dye and the emitted signal of ICG dye was collected by the camera sensor through a micro lens and an 832-nm central wavelength bandpass filter (Semrock, FF01-832–37-25, New York, USA) with bandwidth of 37 nm, which prevented mixing of the strong excitation and weak fluorescence signals at the camera sensor. However, no imaging filters were used for the absorbance and transmission imaging.

**Fig. 11:**
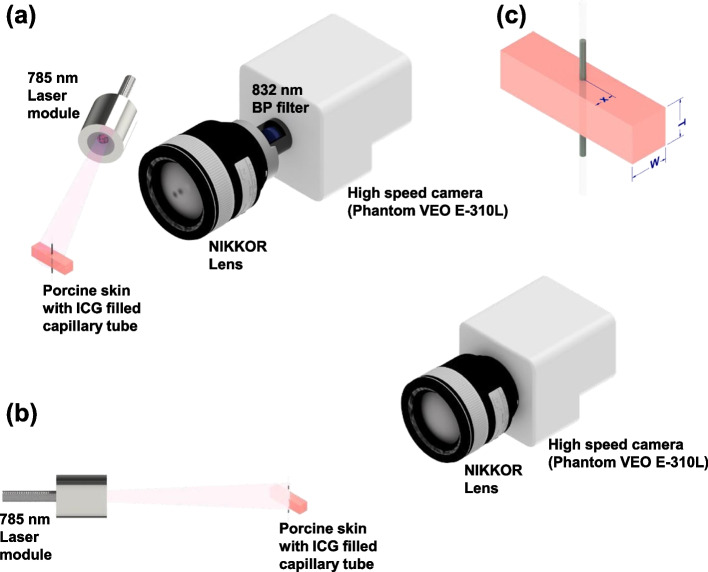
Schematic layout of the experimental setup: (**a**) Fluorescence imaging mode and (**b**) absorbance imaging mode. **c** Annotations of the dimensions shown in Table 1

In absorbance imaging, one of the important optical phenomena is internal reflection which occurs when the incident angle is higher than the critical angle and the light source passes from a medium with higher refractive index to a medium with lower refractive index [86]. The light would be reflected back from the boundary instead of refracting, which can reduce the transmitted signal and cause poor image quality. Therefore, to avoid the internal reflection problem, the laser source was placed in line with the camera at the opposite directions and the samples were placed in between the camera and the laser source, such that the transmittance signal was directly collected by the camera. Figure 11 presents a schematic layout of the experimental setup.

The main objective of the study was to perform high-speed imaging in an ex vivo sample. Therefore, a preliminary study was conducted with ICG solution filled capillaries placed at different depths in tissue, as explained in Ex vivo visualization of NFJI section, to find the optimum imaging condition for ex vivo injection visualization. Images were acquired at exposure times of 10, 50, 100, 250, 500, and 1000 μs and using laser powers of 100, 300, 500, and 700 mW for the fluorescence imaging and 33, 100, and 300 mW for the absorbance imaging. Visualization of the NFJI was performed with the obtained optimum imaging condition.

### Image analysis

The obtained images were analyzed using MATLAB to derive the quantifiers, including SBR, contrast, full width at half maximum (FWHM), and diameter of the capillary tube. The SBR and contrast were computed using Eqs. 1 and 2 as:

1 $$SBR=\frac{Average\;signal\;intensity-Average\;background\;intensity}{RMS\;noise\;approximated\;from\;background}$$

2 $$Contrast=\frac{Average\;signal\;intensity-Average\;background\;intensity}{Average\;background\;intensity}$$

An in-house algorithm was developed for image processing, and the procedure included: histogram equalization of pixel intensities, manual selection of region of interest, segmentation by binarization with a fixed threshold to identify the signal and background, and calculation of parameters. The capillary diameter measurement by full width at half maximum (CDFWHM) was performed by averaging the pixel intensities along all the rows and by obtaining the width at 50% intensity. Thresholding was done by normalizing the image pixel intensity and applying ‘gray threshold’ algorithm in MATLAB. The obtained binarized image was used for measuring the capillary diameter by averaging the length of each pixel row in the image. The obtained measurement is designated as capillary diameter measurement by binarization and thresholding algorithm (CDBTA).

### Ex vivo sample preparation for preliminary study

The ex vivo porcine abdomen skin tissue samples were obtained from a local butcher. For the initial part of the study with capillary tubes, only the epidermis and dermis layers of the skin tissue were considered, and the subcutaneous fat tissue was discarded. The tissues were processed by a freeze–thaw cycle before being sectioned into samples with the required dimensions. Capillary tubes filled with the ICG dye were placed at two positions P1 (x ~ 2 mm), and P2 (x ~ 4 mm) inside the ex vivo samples, where x is the distance of the capillary tube from the imaging edge of the specimen. The corresponding width of the samples was taken as W ~ 2x for each position. Table 1 lists the dimensions of the samples used for each experimental condition.

**Table 1:** Sample dimensions of the ex vivo porcine skin tissue used for imaging. Two positions were considered for the study, where position P1 and P2 signify x ~ 2 mm and x ~ 4 mm, respectively. Standard deviation shows the variation of three sample trials

**Imaging position**	**Clearing time (days)**	**x (mm)**	**W (mm)**	**T (mm)**
**P1**	0	2.55 ± 0.13	4.13 ± 0.09	2.30 ± 0.10
	1	2.42 ± 0.34	3.60 ± 0.33	3.78 ± 0.32
	2	2.67 ± 0.14	4.50 ± 0.40	3.88 ± 0.45
	3	2.69 ± 0.34	3.84 ± 0.33	3.38 ± 0.08
	4	2.73 ± 0.28	3.67 ± 0.15	3.84 ± 0.26
	5	2.65 ± 0.30	3.64 ± 0.29	3.89 ± 0.04
	6	2.54 ± 0.27	4.28 ± 0.23	3.28 ± 0.14
**P2**	0	3.93 ± 0.15	8.00 ± 0.08	2.10 ± 0.10
	1	4.27 ± 0.24	7.26 ± 0.23	3.74 ± 0.10
	2	4.18 ± 0.08	7.41 ± 0.22	4.30 ± 0.26
	3	4.51 ± 0.18	6.95 ± 0.18	2.68 ± 0.20
	4	3.75 ± 0.18	7.37 ± 0.10	3.77 ± 0.16
	5	3.96 ± 0.23	7.28 ± 0.57	3.78 ± 0.17
	6	4.01 ± 0.15	7.61 ± 0.08	3.23 ± 0.04

**Fig. 12:**
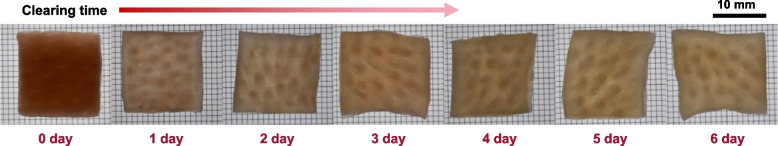
Photographs of the tissue-cleared ex vivo samples. Scale bar = 10 mm

Optical tissue clearing was performed by immersing the samples in 60% TDE (Sigma-Aldrich, St. Louis, MO, USA) in phosphate buffered saline for one to six days. The clearing action of TDE is concentration dependent, and 60% TDE reportedly increases the penetration depth of light [67]. Photographs of the skin tissue samples with varying degrees of clearing are shown in Fig. 12.

### Measurement of light transmission

Light transmission through the cleared and non-cleared skin tissue samples was assessed by measuring the intensity variation of the 785-nm laser light passing through the samples. The light intensity was measured using a handheld laser power and energy meter (Nova Ophir optronic, Jerusalem, Israel). The percentage transmission (%*Transmission*) and absorbance (*A*) values normalized with the skin tissue thickness were calculated as:

3 $$\%Transmission= \frac{(I/{I}_{0})}{T}\times 100$$

4 $$A=\frac{-{log}_{10}(I/{I}_{0})}{T}$$

where *I* is the intensity of the light passing through the sample, *I*_0_ is the reference light intensity measured without the samples, and *t* is the thickness of the sample.

### Mechanical testing of the ex vivo samples

Spherical indentation tests were carried out with universal testing machine (Instron 5567, Norwood, MA, USA) to quantify the Young’s moduli of the cleared and non-cleared ex vivo skin samples. The experiments were conducted with a spherical indenter of 6.35 mm diameter and loading up to 1 N load with a loading rate of 0.5 mm/min. A preload of about 0.1 N was applied prior to the loading of the samples. Studies were conducted with varying clearing duration with three samples each.

The obtained data were processed using MATLAB to deduce the Young’s moduli of the samples. A lowpass filter was applied to the data points to clear the noise. The slope of the linear part of the loading curve was used for computing the reduced Young’s modulus (*E*_*r*_) similar to the method reported in [87]. The Young’s modulus of the skin tissue (*E*_*s*_) was calculated using Eq. 5 as described below:

5 $${E}_{s}=\left(1- {\vartheta }^{2}\right){E}_{r}$$

where $\vartheta$ is the Poisson’s ratio and is assumed as 0.3 for the porcine skin tissue [88].

We also computed the area enclosed between the loading and unloading curves from the indentation test to obtain the total energy and the elastic energy, respectively. The difference between the total and the elastic energies was taken as plastic energy. These parameters were used as quantifiers to compare the fracture toughness parameter of the specimens.

### NFJI visualization

To demonstrate the feasibility of applying the proposed imaging technique for visualizing a NFJI, a spring-powered needle-free jet injector, namely Comfort-in™ (ASTS Enterprises (Aust) Pty Ltd., Burwood, VIC, Australia), was used. The device consisted of a thermoplastic injection chamber and a piston head with a rubber cap. This commercially available device is a mechanically actuated system, wherein the energy from a compressed spring is used for driving the piston to generate a high-velocity microjet through a 150-µm micronozzle, which was used for the injection in this study. Here, 100 µL of ICG in ethanol was injected into the optimally cleared ex vivo porcine skin at a location of 5 mm from the edge, under the optimal fluorescence and absorbance imaging conditions. The 20 mm-thick ex vivo sample consisted of epidermis, dermis, and subcutaneous layers. The subcutaneous tissue had to be considered as the typical final penetration depth of the 100 µL Comfort-in injection was up to the end of dermis layer. In the absence of the subcutaneous layer, the injected fluid penetrated the skin sample and created splashes, which interfered with the imaging. As the total injection time is in the range of milliseconds, the injected fluid does not have a significant diffusion during the penetration and dispersion phase of the injection and may not have an impact on the cleared tissue for the duration of the study. Similar image processing steps as of Image analysis section were used to preserve uniformity in the methodology.

## Supplementary Information

**Additional file 1.**

## Data Availability

Data underlying the results presented in this paper are not publicly available at this time but may be obtained from the authors upon reasonable request.
